# Sharper definitions, blurred lines: The clinical aftermath of the revised antiphospholipid syndrome criteria

**DOI:** 10.1002/hem3.70326

**Published:** 2026-03-22

**Authors:** Lauren E. Merz, Annemarie E. Fogerty

**Affiliations:** ^1^ Division of Hematology/Oncology, Department of Medicine University of Michigan Ann Arbor Michigan United States; ^2^ Division of Hematology/Oncology, Department of Medicine Mass General Brigham Boston Massachusetts United States

The revised criteria for antiphospholipid antibody syndrome (APS) proposed in 2023 by the American College of Rheumatology/European League Against Rheumatism (ACR/EULAR)^1^ have introduced a conundrum in the diagnosis and management of affected patients, particularly those with a history of obstetric APS. An increasingly common challenge is the management of patients who met the 2006 Sapporo criteria for obstetric APS and were managed accordingly with anticoagulation and aspirin in a prior pregnancy, but with the same laboratory and clinical history do not meet the 2023 criteria for a subsequent pregnancy. Whether to continue management based on the old criteria versus to inform the patients they no longer have the disease or indication for active management is an increasingly posed question without a definitive data‐supported answer.

APS is an autoimmune disorder characterized by persistent antiphospholipid antibodies—namely, lupus anticoagulant (LAC), anticardiolipin (aCL), or β2‐glycoprotein‐I (β2GPI) as well as specific vascular or thrombotic complications. The 2006 Sapporo criteria were intended to standardize APS cohorts in research settings but were soon widely adopted as clinical criteria for APS as well. These criteria are dichotomous and relatively simple: one clinical criterion and one laboratory criterion were required for a diagnosis. The 2023 ACR/EULAR criteria introduce a weighted system for both laboratory and clinical criteria and include additional clinical criteria such as thrombocytopenia and livedo reticularis.^1^ The 2006 Sapporo criteria and 2023 ACR/EULAR criteria are compared in Table 1. Given these changes, many patients who had previously been diagnosed with APS no longer meet the revised criteria. We illustrate a common case of reclassification with our expert approach and review of available data:

**Table 1:** Antiphospholipid antibody syndrome (APS) criteria 2023 versus 2006.

	2023 thrombotic APS criteria	2023 weight	2006 thrombotic APS
Clinical	Venous thromboembolism with high‐risk profile	1	One or more documented episodes of arterial, venous, or small vessel thrombosis (excluding superficial venous thrombosis) in any tissue or organ
	Venous thromboembolism without high‐risk profile	3	
	Arterial thrombosis with high cardiovascular profile	2	
	Arterial thrombosis without high cardiovascular profile	4	
	Microvascular involvement suspected	2	
	Microvascular involvement established	5	
	Cardiac valve thickening	2	
	Cardiac valve vegetation	4	
	Thrombocytopenia	2	

	2023 obstetric APS criteria	2023 weight	2006 obstetric APS criteria
Clinical	3+ consecutive miscarriages at <10 weeks and/or early fetal deaths (10–16 weeks)	1	3+ consecutive miscarriages at <10 weeks gestation, or 1+ fetal death >10 weeks gestation, or 1+ premature birth before 34 weeks gestation due to eclampsia, severe pre‐eclampsia, or placental insufficiency
	Fetal death (16–34 weeks) in the absence of pre‐eclampsia with severe features or placental insufficiency	1	
	Pre‐eclampsia or placental insufficiency at <34 weeks with severe features	3	
	Pre‐eclampsia and placental insufficiency at <34 weeks with severe features	4	
Laboratory	Positive lupus anticoagulant ×1	1	Persistent lupus anticoagulant and/or persistent anticardiolipin IgG and/or IgM at medium or high titer and/or persistent beta‐2‐glycoprotein IgG and/or IgM at medium or high titer
	Positive lupus anticoagulant (persistent)	5	
	Persistent moderate or high positive IgM (anticardiolipin or beta‐2‐glycoprotein)	1	
	Persistent moderate positive IgG (anticardiolipin or beta‐2‐glycoprotein)	4	
	Persistent high positive IgG (anticardiolipin or beta‐2‐glycoprotein)	5	
	Persistent high positive IgG (anticardiolipin and beta‐2‐glycoprotein)	7	

*Note*: In the 2023 criteria, a patient must have at least 3 points to meet clinical criteria and at least 3 points to meet laboratory criteria. In the 2006 criteria, one clinical event and one laboratory event are needed for the diagnosis. Persistent refers to positive values at least 12 weeks apart. Moderate titer is ≥40–79 units, and high titer is ≥80 units. Microvascular involvement can include livedo racemosa, livedoid vasculopathy, antiphospholipid antibody nephropathy, pulmonary hemorrhage, myocardial disease, or adrenal disease.

*A 28‐year‐old female has had three spontaneous abortions at 7, 9, and 8 weeks gestation. Fetal karyotype showed no abnormalities in the two losses that had genetic testing performed. She has no history of venous or arterial thrombosis, cardiac valve issues, thrombocytopenia, or microvascular disease. LAC and β2GPI were negative, but aCL IgG was high titer by ELISA twice, 12 weeks apart. The patient is now pregnant at 4 weeks gestation. She asks if she has obstetric APS and what the recommended management is to improve her chances of a live delivery*.

This patient meets the 2006 clinical and laboratory criteria for an obstetric APS diagnosis and continues to meet laboratory criteria by the 2023 definition, but has only 1 of 3 points required to satisfy the 2023 clinical criteria.^1^ Studies have shown that ~15% of patients previously classified as thrombotic APS do not meet the 2023 criteria due to lower antibody titers, IgM‐only positivity, or the presence of exclusion diagnoses.^2^, ^3^ However, nearly a quarter of those reclassified had a thrombotic event during follow‐up.^2^ Additionally, one study showed that only a quarter of patients who met the 2006 obstetric criteria also met the 2023 ACR/EULAR criteria.^4^ In fact, it is impossible to meet clinical criteria on 3+ early losses (<10 weeks gestation) or fetal death (16–34 weeks gestation) alone,^1^ and the majority of reclassifications are due to these clinical events no longer being sufficient.^4^ This is a major departure from the previous diagnosis of obstetric APS.

While APS diagnosis is nuanced, subsequent management is relatively straightforward. Indefinite anticoagulation is recommended for patients diagnosed with thrombotic APS to prevent recurrent thromboembolic events.^5^ In obstetric APS, aspirin and anticoagulation are recommended during pregnancy and 6 weeks postpartum to improve the rate of live delivery.^6^ The safety and efficacy of these treatments have been supported with over 20 years of high‐quality research for patients diagnosed under the 2006 criteria. For example, a meta‐analysis among patients with obstetric APS shows a significantly higher live birth rate with heparin and aspirin compared with aspirin alone (relative risk [RR], 1.29; 95% CI, 1.22–1.35)^7^ while another reported that anticoagulation significantly increased live birth rates (RR, 1.20; 95% CI, 1.09–1.33).^8^

So, what do you recommend for your patient who clearly meets the 2006 criteria for obstetric APS but not the 2023 criteria? First, it is essential to remember that these criteria are intended for research, not for clinical care. Research studies prioritize high specificity while accepting lower sensitivity. In contrast, it is important to prioritize sensitivity in clinical care—especially when the treatment is relatively low‐risk. Randomized controlled studies have shown that only injection site itching or swelling and bruising are significantly increased in women taking aspirin and anticoagulation in pregnancy compared to placebo with no difference in major bleeding.^9^ Second, the research showing a significant benefit of aspirin and anticoagulation was conducted among people with obstetric APS by the 2006 criteria.^7^, ^8^ We currently have no evidence that those reclassified by the 2023 criteria derive no benefit from treatment.

Additionally, the patient experience must be carefully considered when disease criteria are updated. For example, when some patients “lost” their diagnosis of von Willebrand disease after the 2021 update, patient reactions ranged from uncertainty to relief to a sense of loss of validation for their symptoms.^10^ Many patients with obstetric APS are diagnosed in the setting of infertility or recurrent pregnancy losses, which is a particularly vulnerable time. Care must be taken to validate the patient's experience, review available data, and accurately contextualize the patient's journey within it. False hope must be avoided, but limitations or areas of uncertainty in the data should be transparently shared as well. Patients undergoing an evaluation for obstetric APS should be fully informed on which (if any) laboratory and clinical criteria they fulfill by the 2006 and 2023 criteria and the degree of certainty of the diagnosis, so they can actively participate in the discussions around the risks, benefits, and alternatives to treatment with aspirin and anticoagulation for the goal of increasing live birth rate.

In clinical practice, we strongly recommend considering a diagnosis of obstetric APS if a patient meets either the 2006 or 2023 criteria. In fact, we often consider management with aspirin and anticoagulation in a patient who meets laboratory criteria for APS but has only had two unexplained spontaneous abortions at <10 weeks gestation. Rather than rigidly adhering to research criteria intended to prioritize specificity in defining APS, we recommend a more permissive evaluation that balances the intervention's risks (cost, bruising, and itching) with its potential benefits (increasing live birth rate). However, it is also essential to clearly communicate when the likelihood of obstetric APS is low, and treatment would likely only introduce unnecessary cost, bruising, patient discomfort, and false hope. In the case outlined, we would likely recommend treatment with anticoagulation and aspirin after shared decision‐making as she definitively meets the 2006 criteria, despite not meeting the clinical criteria by the 2023 update. Based on studies in obstetric APS that used the 2006 criteria, there is a real possibility of increasing the rate of live birth in her case. We will continue this approach until high‐quality data suggest an alternative approach in these reclassified obstetric APS patients.

Future research efforts should focus on understanding the role of aspirin and anticoagulation in live birth rate among women who have APS by the 2006 criteria but not the 2023 criteria. This may be challenging to complete in a randomized controlled trial setting. Instead, subgroup analysis of the original research supporting the use of aspirin and anticoagulation to improve live birth rate in patients with obstetric APS or observational datasets comparing those who met only the 2006 criteria versus those who met the 2023 criteria should be considered.

The updated 2023 ACR/EULAR APS criteria highlight the tension between research definitions and clinical care as well as gaps in clinical management when we redefine disease. The 2023 update increases the specificity of APS diagnosis and allows for multiple manifestations of clinical disease to be additive rather than binary.^1^ However, these updates also significantly raise the bar for a diagnosis of obstetric APS, which decreases sensitivity and results in uncertainty around best practices for management among women who meet the 2006 criteria but not the 2023 criteria. We advocate for considering both the 2006 and 2023 criteria in determining a diagnosis of obstetric APS, avoiding rigid adherence to research classifications in clinical care, and involving patients in balancing the likelihood of a diagnosis with risks (bruising and injection site discomfort) and potential benefits (increasing live birth rate) of treatment.

## AUTHOR CONTRIBUTIONS

**Lauren E. Merz**: Conceptualization; writing—original draft; writing—review and editing. **Annemarie E. Fogerty**: Writing—original draft; writing—review and editing.

## CONFLICT OF INTEREST STATEMENT

Dr. Merz reports receiving personal fees from Johnson&Johnson and 23andMe.

## FUNDING

This research received no funding.

## Data Availability

Data sharing is not applicable to this article as no datasets were generated or analyzed during the current study.
